# Expression Profiles of Exosomal MicroRNAs from HEV- and HCV-Infected Blood Donors and Patients: A Pilot Study

**DOI:** 10.3390/v12080833

**Published:** 2020-07-30

**Authors:** Karen McGowan, Kenneth J. Simpson, Juraj Petrik

**Affiliations:** 1 Microbiology Research, Development and Innovation, Scottish National Blood Transfusion Service, Edinburgh EH14 4BE, UK; karenmcgowan@nhs.net; 2 Department of Hepatology, Division of Health Sciences, Edinburgh Medical School, Edinburgh EH16 4SA, UK; k.simpson@ed.ac.uk

**Keywords:** HCV, HEV, blood donors, extracellular vesicles, exosomes, microRNAs

## Abstract

Exosomes seem to play an important role in hepatits C virus (HCV) and hepatitis E virus (HEV) infection by shielding their cargo from the host immune responses, with microRNAs being key exosomal components. Little is known about their involvement in a mixed HCV/HEV infection or at the early stages of infection, such as in asymptomatic blood donors (BDs). To obtain preliminary data, we have compared the exosomal microRNA expression profiles in four each of HCV RNA-positive, HEV RNA-positive and negative blood donors and four patients, one of whom was a rare patient with HCV/HEV co-infection. Exosomes were purified from sera by a combination of a precipitation and density gradient centrifugation and exosomal microRNA was analysed using Taqman array cards. Out of 33 deregulated miRNAs, miR-885-5p and miR-365 were upregulated in HCV BDs, miR-627-5p was downregulated in HCV BD and miR-221 was downregulated in HCV patients and BDs. In HEV infection, miR-526b appeared specifically downregulated. Six miRNAs (miR-628-3p, miR-194, miR-151-3p, miR-512-3p, miR-335 and miR-590) indicated a potential involvement in both infections. First time preliminary data on pre- and post-antiviral treatment exosomal microRNA profiles of the HEV/HCV co-infected patient revealed a pool of 77 upregulated and 43 downregulated miRNAs to be further investigated for their potential roles in these viral infections.

## 1. Introduction

Extracellular vesicles (EVs) are released by all types of cells under physiological and pathological conditions as a means of extracellular communication when taken up by the target cells. Three main recognised types of EVs are apoptotic bodies, microvesicles and exosomes (EXs). They differ by size, content and origin in relation to the cellular compartment [1]. Recently, the term small extracellular vesicles has been suggested in preference to exosomes for EVs <200 nm, reflecting the methodical difficulties in producing clean non-overlapping preparations of different EVs [2], but for many authors, exosome is the preferred term. EXs are the most frequently investigated EVs. Their cargo of proteins, lipids and nucleic acid (mRNAs, miRNAs, other small RNAs, sometime DNAs) often differs in composition and quantities from those of the cell of origin, suggesting a selective packaging of exosomal cargo [3,4,5]. While the role of EXs has been most thoroughly investigated in a variety of cancers, a growing number of studies describe the interaction of EX biosynthesis with the pathways and processes of infectious diseases. Increasing evidence points at a number of viruses utilizing EXs as another route of dissemination with the additional benefit of being at least partly shielded from immune responses. Whole virions, infectious genomes, messenger RNAs and viral proteins, some exerting immunomodulatory effects, have all been described among EX cargo [6,7]. EXs appear to play a significant role in liver disease [8]. Hepatitis C virus (HCV) and hepatitis E virus (HEV) are RNA hepatitis viruses belonging to different viral families (Flaviviviridae and Hepeviridae for HCV and HEV, respectively) and differ in their structure, HCV being an enveloped virus, while HEV is non-enveloped. The important role of EXs in HCV infection was first shown by the transmission of replication-competent RNA in cell culture system by RNA-containing exosomes [9]. Another study identified HCV particles in CD81-containing exosomes, described as having a pivotal role in establishing persistent infection through immune evasion [10].

Even more intricate is the role of EXs in viral infection of non-enveloped viruses, such as hepatitis A virus (HAV) and HEV. The ability of HEV to acquire an exosome-like envelope appears to play a role in immune evasion, blurring the difference between enveloped and non-enveloped viruses [11]. HEV virions in faeces are nonenveloped, but those circulating in blood are membrane associated, or quasi-enveloped. Different cell entry mechanisms were suggested for these two types of virions [12], but the role of this envelope in HEV disease pathogenesis in general, and in different clinical phenotypes and patient outcomes in particular, are largely unknown.

Among the most important EX cargo tools of intercellular communication are microRNAs. These small non-coding RNAs are ~22 nucleotides long in their mature form and are involved in the regulation of many genes, predominantly targeting the mRNA 3′ untranslated region (3′UTR), resulting in decreased expression levels of target proteins. Due to the short binding sequences (usually nucleotides 2–7 of mature miRNAs) one miRNA can target a number (on average approximately 100) of different mRNAs, and one mRNA can be targeted by a number of miRNAs [13]. The studies of microRNA involvement in HCV infection by far outnumber those in HEV infection. While the involvement of miRNAs in liver disease has been studied in HCV and HEV patients, there are no data on EXs from individuals with dual infection, since HCV/HEV co-infected patients are extremely rare. We have previously described the elimination of HCV but not HEV RNA in such a patient after sofosbuvir/daclatasvir treatment [14] and used samples in this study to characterise EV cargo, focusing on miRNAs. Moreover, exosomal microRNA expression profiles have not been characterised in HEV RNA-positive blood donors (HEV BDs) and there is only limited information on EX miRNAs in HCV RNA-positive blood donors (HCV BDs). These BDs are asymptomatic while having an active infection, providing an opportunity to look at the early stages of these viral infections. The aim of this study was twofold: (1) to identify potential differences in expression patterns of EX miRNA from negative, HCV, and HEV BDs with those of HCV and HEV patients and (2) to analyse pre- and post-treatment samples of an HEV/HCV co-infected patient. The rationale for analysing exosomal microRNAs rather than microRNAs from total plasma samples follows from the higher variability of the molecules and structures circulating in the blood/plasma/serum since they represent a mix of materials of different origin released by the cells specifically and non-specifically, including by dead/dying cells. Exosomal cargo, on the other hand, appears to be selectively packaged, reflecting the state of the cell of origin and message(s) which needs to be delivered to proximal or distant target cells. The data obtained by the analysis of exosomal molecules provide much more specific information about the current state of the organism/organ/tissue or particular cell population. In addition, these molecules and structures (including virions and their components) are shielded, unlike in plasma where they are unprotected, exposed to catabolic enzymes and components of innate and acquired immune responses, etc. We do not think the plasma microRNA is a relevant control for microRNA from plasma-derived exosomes. Unlike a comparison of the subpopulation of microRNAs packaged into exosomes with that of the cell culture host microRNA population, which can be a useful indicator of selective microRNA packaging, the plasma-derived exosomal microRNAs do not get packaged into exosomes from plasma, but from the various cells they originate from.

## 2. Materials and Methods

### 2.1. Blood Donor and Patient Samples

We analysed samples from each of the four anonymous negative blood donors (DN 1–4), HCV RNA-positive blood donors (DC 1–4) and HEV RNA-positive blood donors (DE 1–4), identified in the course of routine testing (Table 1).

We analysed five patient samples from four patients. There were two samples from patient 1: pre-treatment (P1.1) and post-treatment (P1.2) after 1 month of sofosbuvir/daclatasvir therapy.

Patient 1: A 54-year-old female with HCV cirrhosis, having undergone liver transplant 15 months previously, presented with abnormal liver function tests and was found to be HEV PCR positive. HCV untreated.

Patient 2: A 66-year old female, abnormal liver function tests with chronic HEV infection (PCR positive > 6 months), history of sarcoidosis and hypertension (controlled with amlodipine and bendroflumethazide). Not on any immunosuppressive therapy or previous organ transplant.

Patient 3: A 65-year old female, a liver transplant 1 month previously, HCV and hepatocellular carcinoma (HCC), immunosuppressed with tacrolimus, azathioprin and 20 mg prednisolone.

Patient 4: A 58-year old male, a postrenal transplant 6 years previously, immunosuppressed with Advagraf, on ribavirin 400 mg bd, HEV positive 7 months presampling.

All subjects gave their informed consent for inclusion before they participated in the study. The study was conducted in accordance with the Declaration of Helsinki, and the protocol was approved by the Scottish Medical Research and Ethics Committee (10/MREC/00/74), the West of Scotland Research Ethics Committee (15/WS/0081) and the Scottish National Blood Transfusion Service (SNBTS) Sample Governance Committee.

### 2.2. Exosome Precipitation

EXs were usually isolated from 5 mL of both patient and donor plasma samples using an ExoQuick Plasma prep and Exosome precipitation kit (AMSBIO, Abingdon, UK). Prior to precipitation, samples were centrifuged at 3000× *g* for 15 min to remove cells and cell debris. The plasma samples were pre-treated with thrombin to make them compatible with the kit as per the manufacturer’s instructions, (System Biosciences, Palo Alto, CA, USA). Exosomes were stored at −20 °C until used.

### 2.3. Density Gradient Ultracentrifugation of Enriched EXs

Density gradient ultracentrifugation was used to purify exosomes. A 40% to 10% iodixanol density gradient was prepared using 3× cell suspension medium containing 0.5% (*w*/*v*) NaCL, 10 mM Tricine-NaOH, pH 7.4 and OptiPrep density gradient medium (60% *w*/*v* iodixanol; Sigma-Aldrich, Saint Louis, MO, USA). Samples from exosome precipitations were loaded on top of the gradient and centrifuged for 18 h at 4 °C at 110,000× *g* using the SW 32.1 Ti rotor (Beckman Coulter, Brea, CA, USA). The gradient was then fractionated into 13 approximately 1.2 mL fractions from the bottom of the tube. The refraction index of each fraction was measured. Each fraction was diluted in 9 mL of 3× CSM and pelleted by a second ultracentrifugation step for 3 h at 4 °C at 110,000× *g* using the Type 90 Ti rotor (Beckman Coulter, Brea, CA, USA). Tube positions were marked since the pellets were not readily visible. Supernatants were discarded and pellets were resuspended in 80 µL of elution solution from the Total Exosome RNA and Protein Isolation Kit (Life Technologies Limited, Paisley, UK) by pipetting up and down in the area of the expected pellet, followed by vortexing and storage at −20 °C until used in further applications.

### 2.4. Nanoparticle Tracking Analysis (NTA)

EX concentration and size distribution was determined by NTA using the NanoSight NS300 system (Malvern Technologies, Malvern, UK). Samples (3 µL) were diluted in 1× PBS (Gibco, Waltham, MA, USA) to a suitable concentration as per the manufacturer’s recommendations and analysed under constant flow conditions using a syringe pump. Three 45 s video captures were taken at a camera level of 10 and detection threshold of 6. Data were analysed using NTA 3.1.54 software.

### 2.5. Protein Extraction

EX proteins were extracted from 25 µL of each fraction by adding 50 µL of Exosomal Protein Lysis Buffer P200P (101Bio, Mountain View, CA, USA) and mixing well by pipetting up and down. After incubation for 15 min at 4 °C, the tube was centrifuged for 10 min at 4 °C at 14,000× *g*. The supernatant, containing the extracted EX protein, was transferred to a clean tube on ice. It was stored at −80 °C until required for further downstream assays.

### 2.6. Dot Blots

An activated Immobilion-P Transfer PVDF Membrane (pore size 0.45 µm; Merck KGaA, Darmstadt, Germany) was placed on the top of a TBS-T (20 mM Tris, 150 mM NaCl, 0.05% Tween 20, pH 7.5)-soaked sheet of filter paper (Whatman 3MM, Merck KGaA, Darmstadt, Germany) sitting on the top of one sheet of dry filter paper placed on the top of several layers of paper towels. Two microliters of protein preparations from each gradient fraction were spotted within a pre-marked grid. The membrane was then left to dry for 1.5 h at room temperature.

Membrane incubations and washings were carried out at room temperature with agitation, except the primary antibody incubations. Non-specific sites were blocked with blocking solution (TBS-T with 5% low fat milk powder, Marvel) for 30 min. Primary antibody incubations were in 10 mL of TBS-T containing 5% low-fat milk powder overnight at 4 °C followed by three 10-min washes with TBS-T. Two primary antibodies (anti-TSG1 and anti-MMP2) were biotinylated. A 1-h incubation with secondary Ab-HRP (horseradish peroxidase) for non-biotinylated primary Ab or a streptavidine-HRP conjugate for biotinylated was followed by three 10-min washes or six 5-min washes for secondary Ab and streptavidine-HRP, respectively. Primary antibodies and secondary antibodies (diluted in TBS-T, 5% Marvel): 1: CD9: EXOAB-CD9A-1 CD9 antibody (rabbit anti-human) 1:1000 and goat anti-rabbit HRP secondary antibody 1:20,000; 2: Alix (3A9) mouse Mab (2171S Cell Signalling Technology, Danvers, MA, USA): 1:500 and goat anti-mouse secondary Ab, Fc-specific M4280 (Merck KGaA, Darmstadt, Germany) 1:40,000; 3: TSG101, biotinylated (LSBio, LS-C435416, Seattle, Wa, USA) 1:500 and streptavidine-HRP (Sigma-Aldrich S2438, Saint Louis, MO, USA) 1:1000; 4: MMP2, biotinylated (NovusBio NB200-193B, Cambridge, UK) 1:2000 and streptavidine-HRP 1:1000. A chemiluminiscent signal was detected in a digital imaging system (UVP/Analytik Jena AG, Jena, Germany) after membrane treatment with ECL Plus (Thermo Fisher Scientific, Waltham, MA, USA) according to the manufacturer’s instructions.

### 2.7. Automated Protein Gel Electrophoresis

Two-microliter aliquots of each protein sample extracted from the fractions after density gradient ultracentrifugation were analysed using a TapeStation using P200 ScreenTape (Agilent Technologies, Waldbronn, Germany) and reagents according to the manufacturer’s instructions.

### 2.8. Nucleic Acid Extraction

Nucleic acid was isolated from enriched EX fractions using the Total Exosome RNA and Protein Isolation Kit by Invitrogen (Life Technologies Limited, Paisley, UK). Forty microliters from each fraction were made up to 200 µL using sterile 1× PBS (Gibco, Waltham, MA, USA) prior to nucleic acid isolation. Nucleic acid was eluted in 100 µL nuclease-free water as per the manufacturer’s instructions. Nucleic acid was stored at −20 °C until further use.

### 2.9. microRNA Analysis

RNA extracted from EX-containing density gradient fractions were combined. The volume of combined RNA was reduced to ~20 µL in a SpeedVac and concentration was measured on a Nanodrop (Thermo Fisher Scientific, Waltham, MA, USA). Reverse transcription, preamplification and amplification were done using the TaqMan Array Human MicroRNA Card Set v3.0 (a two-card set containing a total of 384 TaqMan microRNA assays per card). The set enables accurate quantitation of 754 human microRNAs using TaqMan miRNA array cards A and B (Thermo Fisher Scientific, Waltham, MA, USA) following the manufacturer’s instructions. Briefly, two aliquots of each duplicate RNA (~10 ng) were used for cDNA synthesis using primer sets A and B. The miRNA miR-ath-159a (external control not present in mammalian samples) was added to mixes containing Megaplex RT primers A and B prior to reverse transcription, followed by pre-amplification with MegaPlex PreAmp primer A and B mixes (15 cycles) and amplification of 1:4 diluted pre-amplified products on A and B 384-well array cards in 40 cycles using a ViiA7 system (Thermo Fisher Scientific, Waltham, MA, USA).

Data analyses were performed using ExpressionSuite software version 1.1. (Thermo Fisher Scientific, Waltham, MA, USA). Global normalisation and the ath-159a miRNA were used for data normalisation. Only statistically significant (*p* < 0.05) over- or under-expressed miRNAs giving the same outcome under both normalisation regimes between investigated groups were considered relevant.

ExpressionSuite group comparison requires at least two samples per group. This was applicable to the donor sample groups and some of the patients’ groups, but not to pre- and post-treatment samples of the patient co-infected with HEV and HCV or other individual sample comparisons. A cut-off of C_t_ = 35 was chosen and the fold changes of each regulated miRNA species were determined using the 2^−ΔΔCt^ method and averaging the values derived from global and ath-159a normalisations [15].

The microRNAs targeting 3′UTR of HCV and HEV were determined using miRDB interactive software [16].

### 2.10. Statistics

The *p*-values for sample group comparisons from the TaqMan array card experiments were automatically calculated by ExpressionSuite software, v1.1. Student’s *t*-tests and chi-squared tests were applied. Results were considered significant if *p* < 0.05. SPSS version 22 (IBM Corp Armonk, NY, USA) was used for statistical analysis.

## 3. Results and Discussion

### 3.1. Exosome Preparation and Purification

A combination of EX precipitation and density gradient centrifugation was chosen as our method of choice. Our goal was to produce a sufficient amount of material for the characterisation of precipitated EXs and for the extraction of nucleic acid and proteins from each gradient fraction for further analyses. The majority of precipitated EXs were within a range of small EVs (<200 nm), with the exception of HEV BD3, patient 2 and patient 4 (<250 nm) (Table 2). There are other methods of EX preparation, such as differential centrifugation, chromatography-based approaches or affinity purification, which have advantages and disadvantages in relation to the yields and purity of the final preparations. It is fair to say that no method or combination of methods produce completely pure EV subpopulations [2,6,7].

### 3.2. Determination of EX-Containing Fractions

**Figure 1:**
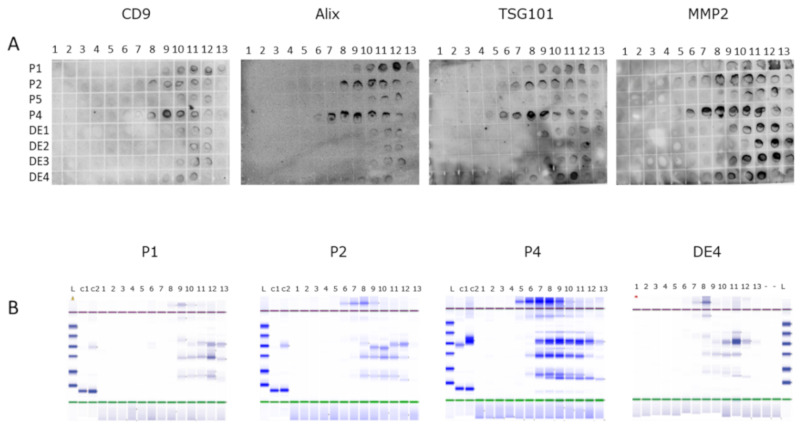
Determination of exosome-containing density gradient fractions of patient (P) and HEV+ blood donor (DE) samples. (**A**) Representative dot blots of proeteins extracted from individual fractions probed with exosome markers antibodies. (**B**) Proteins content of gradient fractions visualized on automated electrophoresis system (Agilent 2200 TapeStation). 1–13: gradient fractions; L: ladder (10–200 kDa); C1-2: lysozyme/BSA control.

Multiple dot blots containing spotted 2 µL aliquots of proteins extracted from density gradient fractions were incubated with antibodies to exosome markers (Figure 1A). In order to confirm the findings from dot blots, another 2 µL aliquot of each protein fraction sample was investigated for the total protein content by automated protein gel electrophoresis (Figure 1B). Both approaches produced similar outcomes as to the fractions with most protein content and the reactivity with exosomal markers. Three or four fractions with the highest signals were selected for the pooling of nucleic acids for further analysis (see Section 3.3).

### 3.3. Exosomal microRNA Analysis

There are far more data published on miRNAs involved in HCV infection than on those involved in HEV infection, but only a limited number of studies investigated exosomal miRNAs. Even less is known about EX miRNAs from HCV or HEV blood donors, since the focus is usually on patient samples. Co-infections may lead to a different pathology but patients with certain co-infections are very rare, as is the case of our HEV/HCV co-infected patient. There were several questions we intended to address. Are there differences in EX miRNA expression patterns between infected and negative donors or between infected patients and infected donors? Which of the differences seem to be HEV specific and/or HCV specific? Which are the miRNAs differentially regulated in a co-infected patient after sofosbuvir/daclatasvir treatment? Since we intended to look at the screening of a larger number of available miRNAs rather than investigating a smaller selection of previously described miRNAs involved in HCV and/or HEV infection, we opted for a pilot study including only a limited number of samples.

#### 3.3.1. Comparison of Blood Donor and Patient Sets of Samples

**Figure 2:**
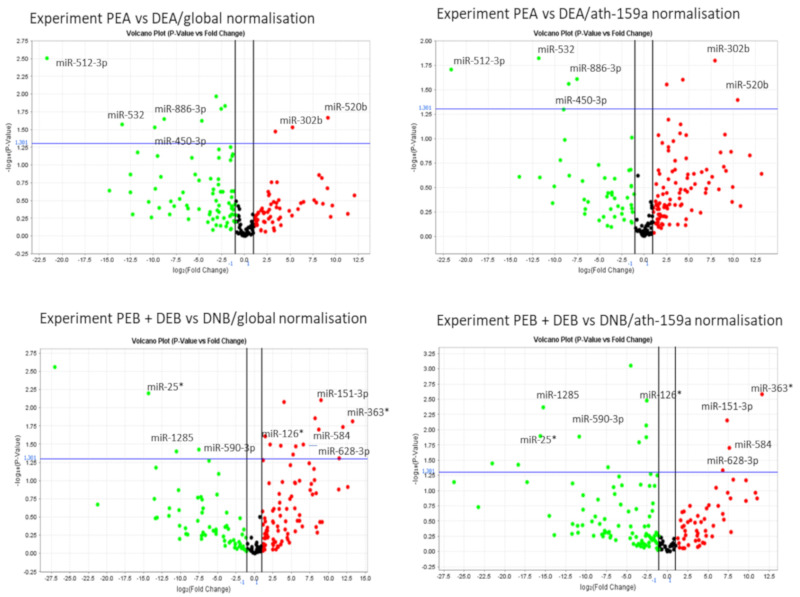
Representative volcano plots generated by ExpressionSuite v1.1. Upper left and right quadrants contain miRNAs with *p* < 0.05. Only miRNAs statistically significant under both normalization regimes are shown and considered as truly significant.

A screening using TaqMan human microRNA array cards (sets of A and B cards) in a series of experiments between different sample groups (Table 1) revealed that 33 microRNAs were differentially expressed in comparisons between blood donor and patient groups, with some repeatedly statistically significant in several comparisons (Table 3). Because we were limited by the available sample volumes, we opted for a stricter selection of miRNAs that were significantly differently regulated. Only miRNAs statistically significant (*p* < 0.05) under both normalisation regimes (global and ath-159a normalisation) were considered truly significant (Table 3, Figure 2).

The analyses revealed certain specific deregulations (Table 3). In HCV samples, the upregulated miRNAs in BDs were miR-885-5p and miR-365 and the downregulated miRNAs were miR-221 (patients and BDs) and miR-627-5p (BDs). In HEV infection, miR-526b appeared to be specifically downregulated. The situation was less clear when more sample groups were combined as a larger group for comparisons, as it is possible that the final significant deregulation may have resulted from a stronger effect in one of the combined groups. This applies to miR-628-3p, miR-194, miR-151-3p, miR-512-3p, miR-335 and miR-590, which are potentially involved in both HCV and HEV infection, as discussed below.

The most frequently differentially expressed microRNA was miR-509-3-5p (five times, Table 3), apparently involved in HCV as well as HEV infection. It was upregulated in HCV BDs and HCV + HEV BDs (all infected blood donors) as compared to negative BDs. It was also upregulated in HCV BD + HCV patients (all HCV-infected samples) against negative BDs, but down-regulated in HCV patients when compared with HCV donors, indicating that the upregulation was rather linked to HCV-infected BDs. It appeared to be upregulated also in HEV infection, since it was up in the HEV BD + HEV patient group as well as in the HCV BDs + HEV BDs when comparing with negative BDs. A strong showing of miR-509-3-5p in this study is intriguing, as there is a lack of published data on its involvement in viral infection. In cancer studies, this microRNA has repeatedly been described as having antiproliferative effects [17].

On the other hand, miR-590-5p was downregulated in both HCV BDs + HCV-infected patients, as well as HEV BDs + HEV patients compared to negative donors, although it has been described as an inducible microRNA with an inhibitory effect on antiviral responses via targeting the IL6 receptor, when investigated in a cell culture system with several viruses, such as influenza A virus, herpes simples virus 1, Sendai virus, Zika virus, vesicular stomatitis virus and human enterovirus 71. A possible explanation for the discrepant results in this study could relate to the different systems used (cell culture versus blood donor and patient plasma samples), as well as the fact that some viruses have an ability to downregulate microRNAs interfering with their replication. HCV and HEV were not investigated in the cell culture study [18]. Similar to miR-590-5p, miR-590-3p was also downregulated in this study, in HEV BDs + HEV patients compared to negative BDs. Since it was downregulated in HCV patients + HEV patients when compared to HCV BDs + HEV BDs, it seems like it may be preferentially downregulated in HEV BDs. Both tumour-promoting and tumour-suppressing effects have been reported for 590-3p in cancer studies, but its role during viral infection has not been identified, yet. Both miR-509-3-5p and miR-590 are strong candidates for future functional studies.

In two experiments, miR 194 was significantly deregulated, being upregulated in HCV BDs and HCV + HEV BDs as compared to negative BDs. Both miR-194 and miR-21 were implicated in the regulation of HCV receptor protein expression and such a role was supported in a study describing miR-194 as hindering HCV entry through targeting one of the HCV receptors, CD81 [19].

In our study, miR-221 was downregulated in HCV patients + BDs compared to negative blood donors. Downregulation was also described during foot and mouth virus infection [20] and respiratory syncytial virus (RSV) persistent infection [21], suggesting a reduced expression in viral infections, which may be interferon induced [22]. There seems to be a mutually negative interaction between HCV and miR-221 since it is one of four microRNAs released from the umbilical cord mesenchymal cell exosomes (together with let-7f, miR-145, miR-199a), largely contributing to the suppression of HCV RNA replication [23]. A different type of regulation leading to miR221/222 cluster overexpression was described for various cancers [24].

In HEV BDs, miR-335 was upregulated when compared with the negative BDs. In the HCV BDs + HEV BDs, miR-335* was upregulated, but not in patients, which was also true for miR-512-3p. In addition, the latter was downregulated in HEV + HCV patients when compared to HCV + HEV BDs, but no published HEV or HCV infection-related data are available for this miRNA. In several viral infections, miR-335 was implicated, which is not surprising considering that it is one of the microRNAs with the highest number of predicted target genes (2544), but no data are available in relation to HEV infection. A downregulation by the HCV NS3 protein was described in a cell culture study [25].

The microRNA 885-5p appears to be a microRNA involved in HCV infection. In this study, it was upregulated in HCV-infected BDs compared to HEV-infected BDs. Upregulation in HCV infection was previously described by El-Diwany and colleagues [26]. It was also among four microRNAs (miR-122, miR-885-5p, miR-221 and miR-22) in a panel demonstrating high diagnostic accuracy for the early detection of HCC in liver cirrhosis HCV patients [27] and one of three circulating microRNA markers of HCV-related chronic liver disease in Egyptian patients, suggesting that miR-885-5p could be a potential marker for advanced liver damage [28].

The fact that hsa-let-7i* was downregulated in this study in HCV BDs + HCV patients compared to negative BDs is in agreement with the conclusions of a paper describing cellular miRNA networks in relation to HCV infection that stated that let-7 (together with miR-25 and miR-130 families) repressed essential HCV co-factors. HCV, in turn, subverted their antiviral actions by dampening their expression [29]. On the other hand, miR-25*, was downregulated in this study in HEV BDs + HEV patients rather than HCV BDs + HEV patients.

In patients infected with HCV of different genotypes, miR-126 was described as downregulated, compared with noninfected individuals [30]. In our study, however, miR-126* was among the miRNAs upregulated in HEV BDs + HEV patients when compared to negative BDs.

The microRNAs deregulated in this study, but without published data linking them to deregulation in HCV or HEV infection, were miR-628-3p, miR-151-3p, miR-526b, miR-1285, miR-520b, miR-302b, miR-627-5p and miR-365, and the following changes all apply to this study:

Both miR-628-3p and miR-151-3p were upregulated in HEV BDs + HEV patients and HEV + HCV BDs when compared to negative BDs, indicating perhaps a closer involvement with HEV infection.

In HEV BDs and HEV BDs + patients, miR-526b appeared to be specifically downregulated when compared to negative BDs.

In HEV BDs + HEV patients, miR-1285 was downregulated (versus negative BDs) and upregulated in HCV patients compared to HCV BDs.

In HCV BDs, miR-627-5p was downregulated compared to negative BDs.

The role of miRNA-365 in viral infection is not well defined. In this study, miR-365 was upregulated in HCV BDs when compared to HEV BDs as well as when compared to the HCV patients, appearing to be specifically upregulated in HCV BDs.

There are several miRNAs deregulated in HEV patients + HEV BDs compared to the negative BDs or in HEV patients compared to HEV BDs without previous reports of involvement in HEV (or HCV) infection (miR-548, miR-584, miR-363, miR-532, miR-450-3p, miR-886-3p, miR-520b and miR-302b), which are candidates for further studies, as are the miRNAs significantly deregulated in a comparison of HEV + HCV patients to HEV + HCV BDs (miR-105, miR-874, miR-876-5p, miR-609 and miR-614). Published data on miRNAs in HEV infection are very limited. Four miRNAs (miR-99a-5p, miR-122-5p, miR-125-5p and miR-192-5p) were deregulated in the serum of the HEV patients with active or chronic infections [31], but in our system, they failed to exhibit a significant deregulation. Published data on the exosomal miRNAs in HEV infection are still missing.

#### 3.3.2. Pre- and Post-Treatment Samples of the HEV/HCV Co-Infected Patient

Another goal of our study was to compare microRNA expression in samples of the patient co-infected with HCV and HEV, who cleared HCV but not HEV RNA after sofosbuvir/daclatasvir treatment [14]. Since the sample group comparison using the ExpressionSuite software requires at least two samples per group, we analysed the pre-treatment (P1.1) and post-treatment (P1.2) samples using the ^∆∆^Ct method to calculate fold changes. In total, 39 miRNAs were downregulated and 71 upregulated by a factor of 2 or more post treatment (Supplementary Table S1). Previously published data linking miRNAs to HCV infection could be found only for around 23 of these miRNAs, no published data link them to HEV infection.

A recently published paper on microRNA changes after direct anti-HCV antiviral treatment (DAA) identified seven significantly reduced microRNAs (miR-122-5p, miR-222-3p, miR-146a, miR-150-5p, miR-30c, miR-378a-3p and miR-20a-5p) [32]. Additionally, miR-122, miR-222 and miR-146a were also downregulated in our sample, but miR-150 and miR-20a were upregulated (although the strand specificity was not shown in the array cards). Unfortunately, the information on the DAA regimen and a dosage was not provided, but while some of the known DAA regimens include sofosbuvir, none of them appears to include daclatasvir, which was used in the treatment of our co-infected patient, making a comparison difficult.

In another DAA study using a combination of ombitasvir/paritaprevir/ritonavir, three miRNAs were significantly upregulated and eight miRNAs were downregulated in the serum of patients 12 weeks after treatment. The majority of deregulated miRNAs were not included on our array cards, but miR-636 was also downregulated in our study. In addition, miR-636 downregulation is not caused by a direct interaction with HCV RNA [33].

It could be expected that some miRNAs known to be induced in HCV infection may be downregulated in the post-treatment sample and vice versa, although the regulatory effects of miRNAs are usually more complex, reflecting their involvement in multiple pathways and processes due to the presence of target sequences in numerous mRNAs. Additionally, the potential effects of HEV in dual infection are difficult to judge, as the published data on miRNAs involved in the HEV infection are largely missing.

Among the miRNAs described as HCV induced was miR-222, which was upregulated in HCV HCC-derived cells [34] and also in HCV HCC patient samples, together with miR-193b [35]. The liver-specific miR-183 was among the miRNAs upregulated in HCC [36], as was miR-22, which was suggested as an early marker of HCC in chronic hepatitis C patients [37]. The miRNA let-7b suppressed HCV replicon activity and downregulated HCV accumulation, most probably by direct interaction with the HCV RNA [38,39]. The exosome-transmitted miR-155, which can inhibit hepatocyte HCV replication, was upregulated in arthritis patients with HCV [40]. An HCV-promoted increase was seen in miR-146a-5p in cell-based models, as well as in samples of HCV patients [41]. All of these miRNAs were downregulated in our post-treatment sample (Supplementary Table S1).

Several miRNAs, which appeared to be reduced in HCV infection, showed an increase after the HCV RNA elimination in our post-treatment sample in this study. In a cell culture study, miR-150 was significantly reduced upon the action of the HCV NS3 protein [26]. In HCV-infected Huh7.5 cells, miR-30b was among the downregulated miRNAs and was and subsequently upregulated following interferon-α treatment [42]. Both miR-17-5p and MAP3K8 expression appeared to be inversely correlated with the response to interferon alpha/ribavirin combination therapy. Both (and together with miR-122) were associated with a poor virologic response [43]. In individuals after they spontaneously cleared HCV, miR-21 was reported to be upregulated [44]. However, in another two studies, miR-21 appeared to be HCV induced [45,46]. In addition to these miRNAs, there were numerous other significantly deregulated miRNAs in both groups without previous published links to HCV or HEV infection. We plan to investigate these miRNAs further, after verification with an independent method.

One of the mechanisms for controlling microRNA levels is a feedback mechanism indicating the increased levels of particular microRNAs as a response to increased concentrations of their target mRNA(s) [47]. Accordingly, some of the microRNAs targeting the HCV 3′UTR may be reduced after HCV RNA elimination in the post-treatment sample (P1.2). We used miRDB interactive software [17] to identify microRNAs targeting the 3′UTRs of 35 HCV and 39 HEV database sequences. A number of the identified microRNAs were not included in the version of the TaqMan array cards we used, as they were discovered more recently (Table 4).

Only two miRNAs out of those shown in Table 4were downregulated in the post-treatment sample. The target sequences for members of microRNA 548 family were identified in 9/35 HCV 3′UTR and 4/39 HEV 3′UTR sequences. One of the members, miR-548c, was downregulated in the post-treatment sample. Another post-treatment reduced miRNA was miR-122, which is considered “liver-specific” and is frequently described as an essential host factor for efficient HCV replication [48,49] and the HCV 5′UTR target sequence is strictly conserved. Similarly, the 3′UTR target sequence is conserved, but apparently only negligibly affecting the HCV replication. While miR-122 was reduced in the post-treatment sample of the HEV/HCV co-infected patient, we did not observe statistically significant differences in our sample group comparisons. We do not know the reason for this, but one explanation could be that the conserved miR-122 target sequence was also described in HEV genomes and appeared to facilitate HEV replication, which is greatly reduced by the inhibition or depletion of miR-122 [50]. This might have skewed the comparisons in the infected samples. Moreover, some other studies reported no or only a weak correlation between the hepatic miR-122 and serum HCV load, and no correlation with the hepatic HCV load [51,52]. The miR-122 levels also appear to be genotype dependent, reported as significantly lower in the serum and exosomes of HCV genotype 1b patients than in controls [53]. We did not genotype our blood donor HCV samples, although the HCV genotype of our co-infected patient was gt3 [14].

This study has several limitations. It was designed as a pilot study to identify the profiles of microRNA expression in five groups of samples and, consequently, the sample numbers are small. While the donor sample sets are homogeneous, the patient samples are more variable. We therefore tried to make data obtained by the ExpressionSuite programme more robust by considering only microRNAs statistically significant under both of the normalisation regimes we used in each comparison—global normalisation and a normalisation based on the external spiked microRNA ath-159a.

Patients with HEV/HCV co-infection are extremely rare. The analysis of dysregulated miRNAs in the pre- and post-treatment samples of this patient was carried out by calculating values using the 2^−ΔΔCt^ method, since the automated analysis with the ExpressionSuite software requires at least two samples per group. While a proportion of dysregulated miRNAs from this pilot study appeared in published studies related to HCV infection and a much smaller proportion related to HEV infection, the remaining miRNAs lack data associating them with these viral infections. The preliminary data obtained in this pilot study will require further verification and follow-up studies.

In summary, we have, for the first time, compared exosomal microRNA expression profiles between HCV BDs, HEV BDs and negative BDs, as well as small numbers of HEV and HCV patients, and obtained preliminary data on several miRNAs apparently closely linked to particular BD and patient groups, such as miR-885-5p and miR-365, which are upregulated in HCV BDs, miR-627-5p, which is downregulated in HCV BDs, and miR-221, which is downregulated in HCV patients and BDs. In HEV infection, miR-526b appears to be specifically downregulated. Another group of six miRNAs (miR-628-3p, miR-194, miR-151-3p, miR-512-3p, miR-335 and miR-590) are potentially involved in HCV as well as HEV infection. We have also obtained, for the first time, preliminary data on the expression profiles of exosomal miRNA from a rare HEV/HCV co-infected patient for pre- and post-antiviral treatment. A number of additional identified deregulated miRNAs without a previously described link to HCV or HEV infection represent an interesting pool for investigations on their potential roles and the role of exosome intercellular communications in these viral infections.

## Figures and Tables

**Table 1:** Sample group definition for data analysis.

Abbreviation	Definition of Groups
DNA	D = donor (D1–4); N = negative; A = Taqman card A
DNB	D = donor (D1–4); N = negative; B = Taqman card B
DEA	D = donor (D1–4); E = HEV-positive; A = Taqman card A
DEB	D = donor (D1–4); E = HEV-positive; B = Taqman card B
DCA	D = donor (D1–4); C = HCV-positive; A = Taqman card A
DCB	D = donor (D1–4); C = HCV-positive; B = Taqman card B
PEA	P = patient (P1.1, 1.2, 2, 4); E = HEV-positive; A = Taqman card A
PEB	P = patient (P1.1, 1.2, 2, 4); E = HEV-positive; B = Taqman card B
PCA	P = patient (P1.1, 3); C = HCV-positive; A = Taqman card A
PCB	P = patient (P1.1, 3); C = HCV-positive; B = Taqman card B

**Table 2:** NanoSight measurements after exosome precipitation.

Sample	Concentration (Particles/mL) +/− SD	Mean Size nm +/− SD (nm)	Merged Data Mean (nm)
HCV BD 1	5.62 × 10^6^ +/− 2.58 × 10^6^	118.8 +/− 10.5	110.3
HCV BD 2	9.20 × 10^8^ +/− 1.53 × 10^6^	146.2 +/− 4.7	144.8
HCV BD 3	3.70 × 10^8^ +/− 2.94 × 10^7^	111.0 +/− 2.3	110.7
HCV BD 4	6.16 × 10^8^ +/− 7.12 × 10^7^	106.3 +/− 2.8	106.4
Negative BD 1	3.16 × 10^7^ +/− 6.85 × 10^6^	168.6 +/− 7.9	169
Negative BD 2	5.84 × 10^8^ +/− 4.43 × 10^7^	100.6 +/− 2.0	100.3
Negative BD 3	9.71 × 10^8^ +/− 2.65 × 10^7^	142.6 +/− 1.2	142.6
Negative BD 4	9.61 × 10^8^ +/− 3.73 × 10^7^	154.9 +/− 2.7	154.9
HEV BD 1	1.08 × 10^7^ +/− 5.3 × 10^6^	115.9 +/− 32.7	144.2
HEV BD 2	1.94 × 10^7^ +/− 6.63 × 10^6^	137.9 +/− 22.0	125.7
HEV BD 3	1.77 × 10^7^ +/− 2.33 × 10^6^	236.9 +/− 53.5	222.9
HEV BD 4	1.40 × 10^7^ +/− 7.93 × 10^5^	175.4 +/− 2.1	175.6
Patient 1.1	2.17 × 10^8^ +/− 2.27 × 10^7^	121.8 +/− 3.8	121.3
Patient 1.2	7.7 × 10^6^ +/− 3.36 × 10^6^	113.7 +/− 75.1	166.6
Patient 2	2.15 × 10^7^ +/− 4.17 × 10^6^	229.3 +/− 30.4	218.4
Patient 3	2.05 × 10^7^ +/− 6.07 × 10^6^	182.9 +/− 24.9	168.2
Patient 4	3.27 × 10^7^ +/− 4.77 × 10^6^	242.1 +/− 101.1	242.1

**Table 3:** Up- and downregulated microRNAs in sample group comparisons.

Experiment	Hsa-miR-	↑Up or ↓Down Regulation	*p* = (Global Normalisation) *	*p* = (ath-159a Normalisation) *
DEA/DNA	526b	↓DEA	0.034	0.020
DEB/DNB	335	↑DEB	0.013	0.022
DCA/DNA	194	↑DCA	0.006	0.002
	509-3-5p	↑DCA	0.010	0.041
	627	↓DCA	0.007	0.026
DCB/DNB	--			
DEA/DCA	885-5p	↑DCA	0.004	0.005
	365	↑DCA	0.012	0.013
	374-5p	↑DCA	0.032	0.036
DEB/DCB	659	↑DCB	0.003	0.003
	32 *	↑DCB	0.019	0.011
DCA + DEA/DNA	194	↑DCA + DEA	0.011	0.009
	509-3-5p	↑DCA + DEA	0.006	0.023
	654-3p	↓DCA + DEA	0.007	0.017
DCB + DEB/DNB	335 *	↑DCB + DEB	0.021	0.017
	628-3p	↑ DCB + DEB	0.017	0.040
	151-3p	↑ DCB + DEB	0.021	0.031
	Let-7i *	↓ DCB + DEB	0.026	0.034
PEA/DEA	512-3p	↓PEA	0.020	0.003
	532	↓PEA	0.015	0.027
	450-3b	↓PEA	0.028	0.030
	886-3p	↓PEA	0.025	0.023
	302b	↑PEA	0.016	0.029
	520b	↑PEA	0.041	0.021
PEB/DEB	335 *	↓PEB	0.019	0.014
PCA/DCA	365	↓PCA	0.045	0.007
	509-3-5p	↓PCA	0.030	0.045
PCB/DCB	1285	↑PCB	0.046	0.045
PEA + DEA/DNA	509-3-5p	↑PEA + DEA	0.002	0.027
	526b	↓PEA + DEA	0.010	0.011
	548a/b	↓PEA + DEA	0.011(b)	0.042(a)
	590-5p	↓PEA + DEA	0.040	0.007
PEB + DEB/DNB	25 *	↓PEB + DEB	0.006	0.013
	1285	↓PEB + DEB	0.040	0.004
	590-3p	↓PEB + DEB	0.038	0.013
	126 *	↑PEB + DEB	0.024	0.003
	628-3p	↑PEB + DEB	0.032	0.047
	584	↑PEB + DEB	0.020	0.020
	151-3p	↑PEB + DEB	0.008	0.007
	363 *	↑PEB + DEB	0.015	0.003
PCA + DCA/DNA	221	↓PCA + DCA	0.013	0.041
	590-5p	↓PCA + DCA	0.027	0.024
	509-3-5p	↑PCA + DCA↓	0.013	0.050
PCB + DCB/DNB	---			
PEA + PCA/DEA + DCA	512-3p	↓PEA + PCA	0.005	0.001
	105	↓PEA + PCA	0.050	0.032
	874	↓PEA + PCA	0.038	0.042
	876-5p	↑PEA + PCA	0.007	0.019
PEB + PCB/DEB + DCB	609	↓PEB + PCB	0.019	0.019
	590-3p	↓PEB + PCB	0.036	0.043
	614	↑PEB + PCB	0.001	0.046

* Only microRNAs statistically significant under both normalisation regimes (global and ath-159a) are shown. For sample group abbreviation, see Table 2.

**Table 4:** The microRNAs with most frequently conserved target sequences in the 3′UTRs of hepatitis C virus (HCV) and hepatitis E virus (HEV) and their amplification status in pre- (P1.1) and post-treatment (P1.2) samples.

microRNAs Targeting HCV 3′UTR *			microRNAs Targeting HEV 3′UTR *		
hsa-miR-	Frequency of Target Sequence **	Amplification in P1.1 and P1.2	hsa-miR-	Frequency of Target Sequence **	Amplification in P1.1 and P1.2
122	33	↓in P1.2	298	9	Not amplified in either
505-5p	17	Failed to amplify in P1.2. Not included	539-5p	6	539 no significant change
651-3p	14	Not amplified in either	328-5p	5	328 no significant change
548 family	9	548c ↓ in P1.2	548 family	4	548c ↓ in P1.2
1252-5p	8	1252 no significant change			
939-5p	4	939 no significant change			
509-3/5p	4	No significant change			

* Only microRNAs included in TaqMan array cards A and B are shown. ** Those with four or more targets among the 35 HCV and 39 HEV complete genomic sequences are shown. Accession numbers of the analysed sequences are shown in Supplementary Table S2.

## Supplementary Materials

The following are available online at https://www.mdpi.com/1999-4915/12/8/833/s1. Table S1: Differentially expressed miRNAs in the pre-treatment (P1) and post-treatment (P2) samples of a patient co-infected with HEV and HCV; Table S2: Accession numbers of sequences analysed for the presence of miRNA target sequences in HCV and HEV 3′UTR.

## Abbreviations

BD: blood donor; EV: extracellular vesicle; EX: exosome; HCC: hepatocellular carcinoma; HCV: hepatitis C virus; HEV: hepatitis E virus; HCV BD: HCV RNA-positive blood donor; HEV BD: HEV RNA-positive blood donor; miRNA: microRNA; PCR: polymerase chain reaction.
