# Supplementary material for: Expression Profiles of Exosomal MicroRNAs from HEV- and HCV-Infected Blood Donors and Patients: A Pilot Study
Source: Viruses. 2020 Jul 30;12(8):833. doi: 10.3390/v12080833 (PMC7472156; doi:10.3390/v12080833)
Supplement: Supplementary file 1 [file viruses-12-00833-s001.zip › Supplementary Table S2.docx]

**Table S2.** Accession numbers of sequences analysed for presence of miRNA target sequences in HCV and HEV 3’UTR.

| **Accession numbers of analysed 3’UTR HCV sequences** | **Accession numbers of analysed 3’UTR HEV sequences** |
| --- | --- |
| D85016.1, D85017.1, DQ329235.1, JN836949.1, D63922.1, AB677527.1, AF333324.1, AB049101.1, AB049094, NC_009823, AB558135.2, U45476.1, NC_038882.1, AB795432.3, KM102764.1, NC_030791.1, NC_009824.1, NC_009827.1, NC_009825.1, MH590700.1, KY283130.1, KM587622.1, EF108306.2, EU781826.1, AB828701.1, AB690460, AB691596.1, FN666428.2, GU133617.1, AM910652.2, AJ851228.1, D84265.2, D84263.2, D84262.2, EU835526 | MH504163.1, MH504161.1, AY220474.1, MH504162.1, MH504158.1, MH504153.1, MH504151.1, MH504146.1, MH504142.1, MH504140.1, MH504136.1, MH504133.1, MH504131.1, MH504128.1, MH504124.1, JQ655736.1, KC163335.1, AB425830.1, LC176493.1, LC126332.1, AB437318.1, AB369687.1, AB291960.1, AB850879.1, KX172133.1, KU980235.1, AB291961.1, FJ653660.1, AB193177.1, MH450031.1, MH450022.1, KY232313.1, KC492825.1, AB291965.1, AB099347.1, AB291964.1, KT581443.1, AB593690.1, KT581448.1 |
